# Developing a Shared Decision-Making Model for Diabetic Patients: A Qualitative Study

**DOI:** 10.31661/gmj.v10i0.1735

**Published:** 2020-06-28

**Authors:** Khorshid Vaskouei-Eshkevarei, Kamran Hajinabi, Leila Riahi, Mohammadreza Maleki

**Affiliations:** ^1^Department of Health Services Administration, Science and Research Branch, Islamic Azad University, Tehran, Iran; ^2^Department of Health Services Management, School of Health Management and Information Sciences, Iran University of Medical Sciences, Tehran, Iran

**Keywords:** Decision Making, Shared, Patient Participation, Models, Theoretical

## Abstract

**Background:**

Patient participation in healthcare leads to increased satisfaction and trust, reduction of anxiety, and a better understanding of patients’ needs. The components of shared decision-making (SDM) are well documented in the literature. The purpose of this study was to design an SDM model for diabetic patients.

**Materials and Methods:**

This qualitative content analysis study was performed in three steps. First, a descriptive comparative study was conducted using the widely-used George Brady method. Next, the perceptions of participants (both physicians and patients) were collected via interview and in focus group discussions (FGDs). Content analysis was used to categorize the comments made by participants. In the final step, the model of SDM for diabetic patients was designed based on expert panel discussions.

**Results:**

Twelve components were extracted from the comparative study. Two themes and six sub-themes were extracted from data resulting from physicians’ interviews, and two themes and ten sub-themes were extracted from data resulting from the FGDs involving patients. The model of SDM for diabetic patients was designed in light of three concepts; practitioners’ behavior, participatory decision-making process, and patients’ autonomy.

**Conclusion:**

This model was valuable because it recognizes the process of SDM in the context of Iran. The model’s main purpose was to help choose optimum strategies for the care of diabetic patients within the health sector.

## Introduction

Shared decision-making (SDM) is a process in which patients and clinicians make decisions and reach consensus through cooperative communications. In SDM, patients and physicians share information about the goals they hope to achieve and the tasks necessary to meet such goals[1]. Patient participation in decision-making is a process that accepts patients as stakeholders, counselors, and decision-makers [2]. Increasing patient participation in healthcare policies can lead to improved patient satisfaction, quality and safety, cost savings, and community health outcomes [3,4]. It is often reported that the involvement of other providers of healthcare services, other than doctors, is important in decision-making [5,6]. Ben-Zacharia et al. (2018) argue that greater collaborative decision-making between patients and physicians would lead to better follow-up treatment for multiple sclerosis (MS) [7].A preventive approach involves collaborative decision-making that is based on patient preferences, training, and engagement. Providing reliable and accurate sources of information is essential to improving patient participation [7].Nakayama (2018) asserted that respect for patient values has increasingly been recognized in evidence-based medicine, and joint decision-making between patients and doctors has been considered [1]. Tinelli et al. (2017) showed that although patients tend to participate in collaborative decision-making, such processes and counseling are not desirable [8]. Serrano et al. stated that collaborative decision-making creates an empathy conversation between patients and physicians that combines the best available evidence with values, preferences, and fields [9]. Kolovos et al. stated that organizational structure, patients’ knowledge, and willingness to participate in treatment are vital [10].In Iran, the Charter of Patients' Rights was drafted and announced by the Ministry of Health and Medical Education in 2000, in which the patient's participation in decision-making is considered as one of the main rights of patients. Therefore, further research is needed to design models to describe patients' participation in decision-making. The purpose of this study was to design a model for shared decision making in Iran.

## Materials and Methods

 This study was a qualitative content analysis following a constructivist approach. It was conducted in three steps: a comparative study, qualitative study, and model development. These three steps are explained in more detail below.

## Comparative Study

 This descriptive comparative study was conducted using the widely-used George F. Brady method[11]. To acquire the required information, a wide search was carried out of articles published in English and Farsi during 2017 - 2018. The search was conducted by using the relevant keywords include “SDM,” “participation,” “diabetic patients,” and “descriptive comparative study.” All searches were in PubMed, Medline, Scopus, Google Scholar, the Scientific Information Database (SID), and Iran’s Medex database. Using this model, information gathered, stages of description, interpretation, proximity, and comparison were all analyzed.

##  Qualitative Study

 In this step, the qualitative study was carried out using semi-structured interviews of eight physicians (internists and endocrinology specialists) and focus group discussions (FGDs) with 24 patients. Informed consent was obtained from all 24 patients.

##  Interview

 CThe interviewer was a Ph.D. student in the management of health services (the first author). The interviews were initiated with the general question of “how do you contribute to the treatment of patients?” Some more probing questions were also asked. The interviewer stimulated the participants to give more descriptions of the areas of interest. The interviews were conducted in a quiet room and recorded using a tape recorder. Each interview lasted between 35 and 65 minutes. The interview guide was semi-structured and used open-ended prompt questions. The interviews were almost immediately transcribed and analyzed. Participants followed a purposive sampling method. Sampling and data collection continued until saturation of data, which was determined through almost immediate analysis of the data after the transcription of each interview. The data were analyzed using the qualitative content analysis approach.

##  FGDs

 Participants were involved in seven FGDs that used purposive sampling. E-mail invitations were sent to participate in the focus groups describing the purpose of the study. None of the participants refused the request to participate. Participants were all from the Tehran University of Medical Sciences’ educational hospitals. We collected views of the patients in an open-ended way. Each FGD meeting lasted around 90 minutes. Focus groups were conducted by moderators using the discussion guide. The focus groups were audiotaped and then transcribed. We asked open-ended questions to explore participants’ perceptions of SDM.

##  Design of the Model

At this stage, the nominal group technique (NGT) was used to structure each expert panel discussion. The participants in this phase were eight internists. In this part of the study, the factors affecting the patients’ participation were extracted and finalized based on the results of the two previous stages of the study.Then the patients’ participation areas were determined during the decision-making, and eventually, the conceptual model of the patients’ participation was finalized.

##  Ethical Considerations

 This study was approved by the Ethical Committee of the Science and Research Branch, Islamic Azad University (approval code: IR.IAU.SRB.REC.1396.69).

## Results

 1.Comparative Study ResultsThe results of a comparative study based on the Brady model are summarized in four stages: descriptions, interpretation, neighborhoods, and comparison. The results of the description phases are shown in (Table-1).

1.1. Interpretation and Neighborhood Phases Based on the Brady ModelDuring the interpretation stage, the research team members analyzed the similarities and differences for each model. During the neighboring stages, patient involvement patterns in the SDM are summarized in (Table-2).

1.2. Comparative Phases Based on the Brady Model In this step, to answer the research question, the percentage of repetition of each component was extracted in the various patterns that are shown in (Table-3).

2. Qualitative Study Results

2.1. Interview of Physician Participants 

2.1.1. Demographic Characteristics in the Interview Phases The distribution of the sampling applied criteria is presented in (Table-4).

2.1.2. Results of Qualitative Analysis in Physician Interview Two themes and six sub-themes were extracted and are summarized in (Table-5).

3.Design of the Conceptual Model 

**Figure-1:**
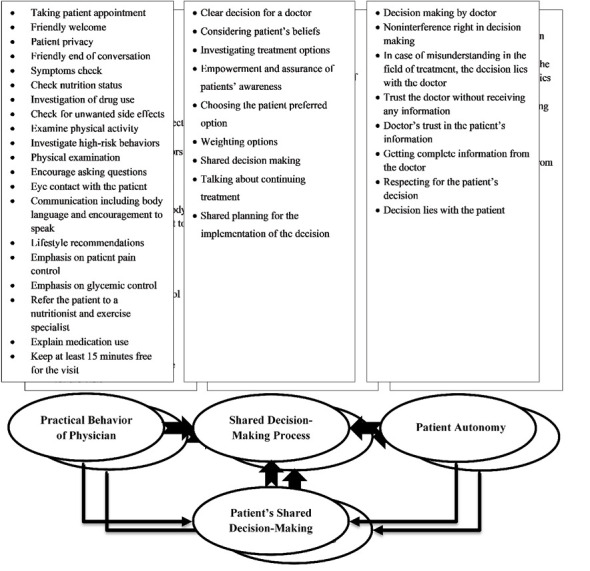
The conceptual model of shared decision-making

In the expert panels’ discussions, the model was designed based on the results of comparative study and qualitative content analysis. The schematic conceptual framework of the research to be conducted in this study is shown in (Figure-1).

## Discussion

Based on the results of this study, the conceptual model of SDM was designed. This model describes the factors that influence the patient’s participation in the decision-making process. Our findings are consistent with those of other studies by Charles et al., the Longtin model, or the conceptual model of the World Health Organization [16]. Based on our results, the practical behavior of physicians has been recognized as one of the key factors that influence the process of SDM. The findings of this study are consistent with those of Ben-Zacharia et al.[7] showed that constructive interaction between physician and patient is vital to effective SDM. These results also provide further support for the hypothesis that respect for patients’ rights and effective communication with patients improves SDM in health [26]. 

The results of this study have shown that the physicians’ knowledge and positive attitude toward participating in decision-making are necessary, but not of themselves enough. Indeed, empowerment of the patient, the perceived ability of the patient to self-manage him or herself, selection of decision-making patterns by physicians such as descriptive, analytical, educational, advisory, and patient-centered care, and support of patient autonomy are all very important [3,27,28]. The results of this study have demonstrated that, in addition to other factors, the duration of the consultation affects the satisfaction of the patients and promotes effective SDM. Indeed, not allocating sufficient time for a patient visit leads to a reduction in patient SDM [29].

Based on our results, the SDM process with nine stages was another concept that is essential to SDM. Considering the patient’s beliefs and values in making decisions, as well as patient preferences, leads to continuing effective treatment [26]. Important outcomes of patient participation in decision-making include reducing the fear and stress of the patient, improving the quality of life, and thereby increasing patient satisfaction [30,31]. Another concept in our SDM model is patient autonomy. This construct ranges from a patient’s unwillingness to participate in decision-making at all to a ready willingness to make decisions. Patient preferences in collaborative decision-making and patient choices lead to increased self-esteem. This, in turn, encourages the possible interaction between the doctor and the patient through the exchange of information and revealing individual preferences [30]. Patient control of decision-making is one of the dimensions of active participation in care that is defined in long-standing notions such as those of informed consent or sharing of power and responsibility. Encouraging the patient to engage in counseling actively and engaging the patient in making decisions underlines the importance of helping the patient make informed choices [31]. From the results of this study, we propose a practical model to be used in the health system of the Islamic Republic of Iran. This study provides a model for promoting and modifying the physician’s practical behavior concerning patient participation in treatment decision-making. This model can also be used to educate and empower both patients and physicians in Iranian hospitals and other treatment centers. Considering our results, one of the most important aspects of patient participation in treatment decision-making is patient preference and autonomy. One of the ways to achieve those is to improve the culture and improve patients’ attitudes towards their participation in decision-making. For the qualitative results, the two features of credibility and confirmability were used to confirm the accuracy of the data and findings in our study. The credibility of the findings was confirmed using member-checking. To increase the confirmability of the findings, external peer checking was also used. One limitation of the study was that only patients willing to give informed consent were included. Another limitation was the impact of a culture that holds that the physician is always the primary decision-maker in the treatment of patients.

## Conclusion

 In this research, the conceptual model of SDM of diabetic patients is designed in the Iranian healthcare system. Patients SDM in this model include three domains: the practical behavior of physicians, the SDM process, and patient autonomy. This effective shared design leads to reducing the patients’ fear and stress, improving their quality of life, and increasing their satisfaction.

## Acknowledgment

 This study was derived from a doctoral thesis about health services management at Azad University, Tehran, Iran (code: IR.IAU.SRB.REC.1396.69). All those who collaborated in the research are warmly appreciated.

## Conflict of Interest

 None.

**Table 1:** The Main Concepts of Common Models about Patient Shared Decision-Making.

**Scientist**	**The main concepts of common models**
**Peter (2012)**	1-Patient characteristics: Elderly - Inactive - No need for more information - Acceptance of physician decisions. Practitioner style: Paternalism. 2-Patient Characteristics: Willingness to Get a Doctor - Ability to Understand Medical Information - Lack of Patient Opinions for Decision Making - Trust in the Physician. Practitioner's style: Distinguished style 3-Patient characteristics: Young patient - University educated - Need to be persuaded before agreeing to any treatment - Believing he or she has the right to challenge the physician. The patient has an active role to play in decision making. Practitioner's style: Collaborative Approach 4-Patient characteristics: Low information - unwillingness to accept medical information - inability to understand medical knowledge. Practitioner's style: Guided Style [12].
**Durand et al. (2015)**	Focus on cultural change The doctor chooses a treatment or screening without any benefits or disadvantages. The risks and benefits of existing choices and patient preferences are considered Positive and negative consequences for patients are discussed [13].
**Engström (2014)**	Factors influencing patient participation in decision making are discussed. The relationship between empowerment factors such as education and service systems, patient participation and outcome implications such as patient satisfaction, cost savings and health outcomes have been studied [14].
**Elwyn et al. (2012)**	Attention to factors related to patients and factors associated with service providers A review of our range of patient preferences and informed patient preferences Paying attention to the correct and widespread support for decision making [15].
**Longtin et al. (2010)**	Conceptual model effective factors in patient shared decision making Attention to effective factors in patient participation in treatment decision-making (factors associated with patients, related factors of service providers) Attention to demographic and field variables Paying attention to community support of the patient Attention to culture [16].
**Talcott Parsons**	The classical theory in the field of sociology, centered on the social system Physician and Patient Relations: The role of the doctor as a "professional person" and the role of the patient as "patient"[17].
**Freidson (1970)**	Putting our fundamental difference between the rights and responsibilities of patients Different roles of doctor and patient Socioeconomic status of the patient. There is a conflict of interest between the doctor and the patient. The ownership of medical knowledge determines the relationship between the doctor and the patient [18].
**Roter and Hall (1992)**	Pay attention to the variable range of physician and patient communication The focus is on patient control over the physician and patient communication process [19].
**Charles et al. (1997)**	Intermediate approach in decision making Attention to the sharing of information along with informed and professional decision making Pay attention to the patient's point of view and physician [20].
**Grol and Wensing (2004)**	Attention to changes in order to implement to the sustainable care Paying attention to community support of the patient Attention to patient education and community Paying attention to communication and providing effective feedback [21].
**Siminoff et al. (2005)**	Conceptual Concepts of Behavioral Decisions and Ethical Concepts The physician-patient relationship that depends on the following factors: • The context of creating a relationship • Communication atmosphere created by physician and patient • Treatment preferences from the perspective of the physician and the patient • Decision making is a social process • People interact with each other, • Exchange information; • Make preferences, • Choose a route [22].
**Emanuel et al. (1992)**	Four types of physician-patient relationship: 1. Educational: Patient values are clear. The patient is given the necessary information. The patient has the right to choose. The physician has the technical expertise and competence. 2. Descriptive: Patient values are unclear and need clarification. Physician actions include: giving information to the patient, performing selective patient interventions, giving advice to the patient. 3. Advice: The patient expresses his or her values; the patient's selective interventions are implemented. As a friend and teacher, the doctor helps to empower the patient, choosing the right treatment for the patient 4. Paternity: Patient value is determined by the patient and the physician. The doctor treats the patient regardless of his or her preferences.[23].
**Elwyn et al. (2017)**	Dialogue with the patient takes place in three stages: 1. Form the team and talk about choosing the goals 2. Talk about options and explain options using rules 3. Talk about decision-making to specific and prioritized priorities and decide on the priority. Active listening and patient consultation are the requirements of this template [24].
**Légaré et al. (2010)**	Attention to the social norms, organizational practices and procedures, and organizational structure Providing patient and family counseling leads to a better understanding of the patient The patient interacts with the team. Decision-making involves: changing information, considering patient preferences, reviewing treatment options, choosing the best option, implementing the decision, ultimately reviewing the consequences of the decision, and choosing a new one if needed [25].

**Table 2:** Matrix of Components Affecting Shared Decision-Making.

**Components**	**Légaré**	**Elwyn**	**Emanuel**	**Siminoff**	**Grol and Wensing**	**Charles**	**Roter and Hall**	**Freidson**	**Talcott Parsons**	**Longtien**	**Elwyn**	**Engström**	**Durand**	**Peter**
**Background**	+	+	+	+	+	+	+	+	+	+	+	+	+	+
**The relationship between the doctor and the patient**	+	+	+	+	+	+	+	+	+	+	+	+	+	+
**Patient **	+	+	+	+	+	+	+	+	+	+	+	+	+	+
**Practical behaviors of the doctorn**	+	+	-	-	-	-	+	+	+	-	+	+	-	+
**Patient empowermentn**	+	+	-	-	-	+	+	+	+	-	-	+	+	+
**The beliefs and values of the patients**	+	-	-	+	-	+	-	+	+	-	-	+	+	+
**Treatment optionsg **	+	+	-	+	-	-	+	+	-	-	-	+	-	-
**Control of the patient over decision making **	+	+	+	+	+	-	-	+	+	+	-	-	-	+
**The outcomes of participation **	+	+	+	+	+	-	+	-	-	-	-	-	-	+
**The patient characteristic**	+	-	-	-	+	-	+	-	-	+	-	-	-	+
** The role of the physician in society**	-	-	-	-	-	+	+	-	-	-	+	+	-	-
** Support by the physician for decision-making in community**	-	-	-	+	-	-	-	-	-	+	-	-	-	-

**Table 3:** The Percentage of Each of the Components of Shared Decision-Making.

** No.**	** Components**	**Percent **
1	** Background**	100
2	**The relationship between the doctor and the patient **	100
3	**Patient preferences **	100
4	**Practical behaviors of the doctor **	64
4	**Patient empowerment**	64
5	**The beliefs and values of the patients **	57
6	**Treatment options **	57
7	**Control of the patient over decision making **	50
8	**The outcomes of participation **	43
9	**The patient characteristic **	36
10	**The role of physician in society **	21
11	**Support by the physician for decision-making in community **	21

**Table 4:** Demographic Characteristics of the Physicians Participating in the Interview Phase.

**No. **	**Specialty **	** Age (Gender) **
1	Endocrinologist	49 (F)
2	Endocrinologist	45 (F)
3	Internal specialist	39 (M)
4	Internal specialist	42 (F)
5	Endocrinologist	55 (M)
6	Endocrinologist	49 (M)
7	Endocrinologist	53 (M)
8	Endocrinologist	48 (F)

**F:** Female; **M:** Male.

**Table 5:** Themes and Subthemes and Physician Quotes Extracted from Interview Phase.

**Theme **	**Subtheme **	**Participant quot **
** Attitudes toward participation in decision-makingn **	Definition of shared decision-making	G3: “Before anyone, the patient needs to understand the importance of healing. We know that everyone has to think for himself.”
Terms of decision	G1: “Look, We need an experienced nurse at our diabetes clinics who can explain a few things to our patients.”	undefined
Level of patient participation in decision-making	G5: “The final decision about the patient is usually the responsibility of the doctor because he has information that the patient doesn’t have.”	undefined
**Experiences about participation in decision-making **	Participation time in decision-making	G3: “If we have enough time, then we involve the patient in the decisions, but if we do not have time, then we should make the best decision for the patient.”
Patient characteristics for participation in decision-making	G1: “Our patients are a heterogeneous group, and I think depending on the educational level and socioeconomic status of patients.”	undefined
Various treatment options	G2: “I had a patient who was not controlled by sugar, with two or three medicines, and A1C was high. I suggested that he should take insulin, but he refused, then I ordered another two taps, and he followed the diet for three months. I did not expect sugar to be controlled, but when it was checked, the sugars were controlling.”	undefined
